# The Cross-Talk Between EGFR and E-Cadherin

**DOI:** 10.3389/fcell.2021.828673

**Published:** 2022-01-20

**Authors:** Miguel Ramírez Moreno, Natalia A. Bulgakova

**Affiliations:** School of Biosciences and Bateson Centre, The University of Sheffield, Sheffield, United Kingdom

**Keywords:** adhesion, morphogenesis, cancer, signalling, epithelia

## Abstract

Epidermal growth factor receptor (EGFR) and adhesion protein E-cadherin are major regulators of proliferation and differentiation in epithelial cells. Consistently, defects in both EGFR and E-cadherin-mediated intercellular adhesion are linked to various malignancies. These defects in either are further exacerbated by the reciprocal interactions between the two transmembrane proteins. On the one hand, EGFR can destabilize E-cadherin adhesion by increasing E-cadherin endocytosis, modifying its interactions with cytoskeleton and decreasing its expression, thus promoting tumorigenesis. On the other hand, E-cadherin regulates EGFR localization and tunes its activity. As a result, loss and mutations of E-cadherin promote cancer cell invasion due to uncontrolled activation of EGFR, which displays enhanced surface motility and changes in endocytosis. In this minireview, we discuss the molecular and cellular mechanisms of the cross-talk between E-cadherin and EGFR, highlighting emerging evidence for the role of endocytosis in this feedback, as well as its relevance to tissue morphogenesis, homeostasis and cancer progression.

## Introduction

Few components are as determining for cell behaviour and fate as Epidermal Growth Factor Receptor (EGFR), which mediates such diverse processes as cell proliferation, survival, growth and differentiation (Wee and Wang, 2017). EGFR is a member of the ErbB family, which is respectively a part of the receptor tyrosine kinase superfamily (Herbst, 2004; Hynes and MacDonald, 2009). Downstream, it transduces multiple signalling pathways, most notably Ras/MAPK, PI3K/AKT/mTOR and PLC/PKC signalling (Oda et al., 2005; Wee and Wang, 2017). Cancer cells often display upregulation of the EGFR signalling or receptor overexpression (Rowinsky, 2004; Guo et al., 2015; Wee and Wang, 2017; Sigismund et al., 2018). This highlights the importance of understanding the regulation and function of the EGFR signalling for novel cancer therapies (Yarden, 2001; Rowinsky, 2004; Vecchione et al., 2011; Sigismund et al., 2018).

Another important component controlling interactions between cells and with their environment is cell adhesion, mediated by Cell Adhesion Molecules (CAMs) (Gumbiner, 1996; Chothia and Jones, 1997). CAMs perform structural functions by linking extracellular space to the cystoskeleton inside cells (Parsons et al., 2010). However, rather than just gluing cells, adhesion also acts as a sensory tool to gather informational cues from the neighbouring cells and substrate (Geiger et al., 2009; Hamidi and Ivaska, 2021). Among the CAMs, E-cadherin is a the major component of the Adherens Junctions in epithelial cells, responsible for cell-cell adhesion (Takeichi, 1977; Chothia and Jones, 1997; Halbleib and Nelson, 2006).

Increasing evidence demonstrates interactions between the EGFR signalling and E-cadherin-mediated cell-cell adhesion. An inverse correlation between levels of EGFR and E-cadherin was reported in various cancers including endometrial carcinoma, cholangiocarcinoma, head and neck squamous cell carcinoma, and breast carcinoma to name a few (Jones et al., 1996; Zuo et al., 2011; Clapéron et al., 2014; Yang et al., 2014). Here we summarize the existing data on these interactions and highlight the major remaining gaps.

## Overview of EGFR Regulation

**FIGURE 1:**
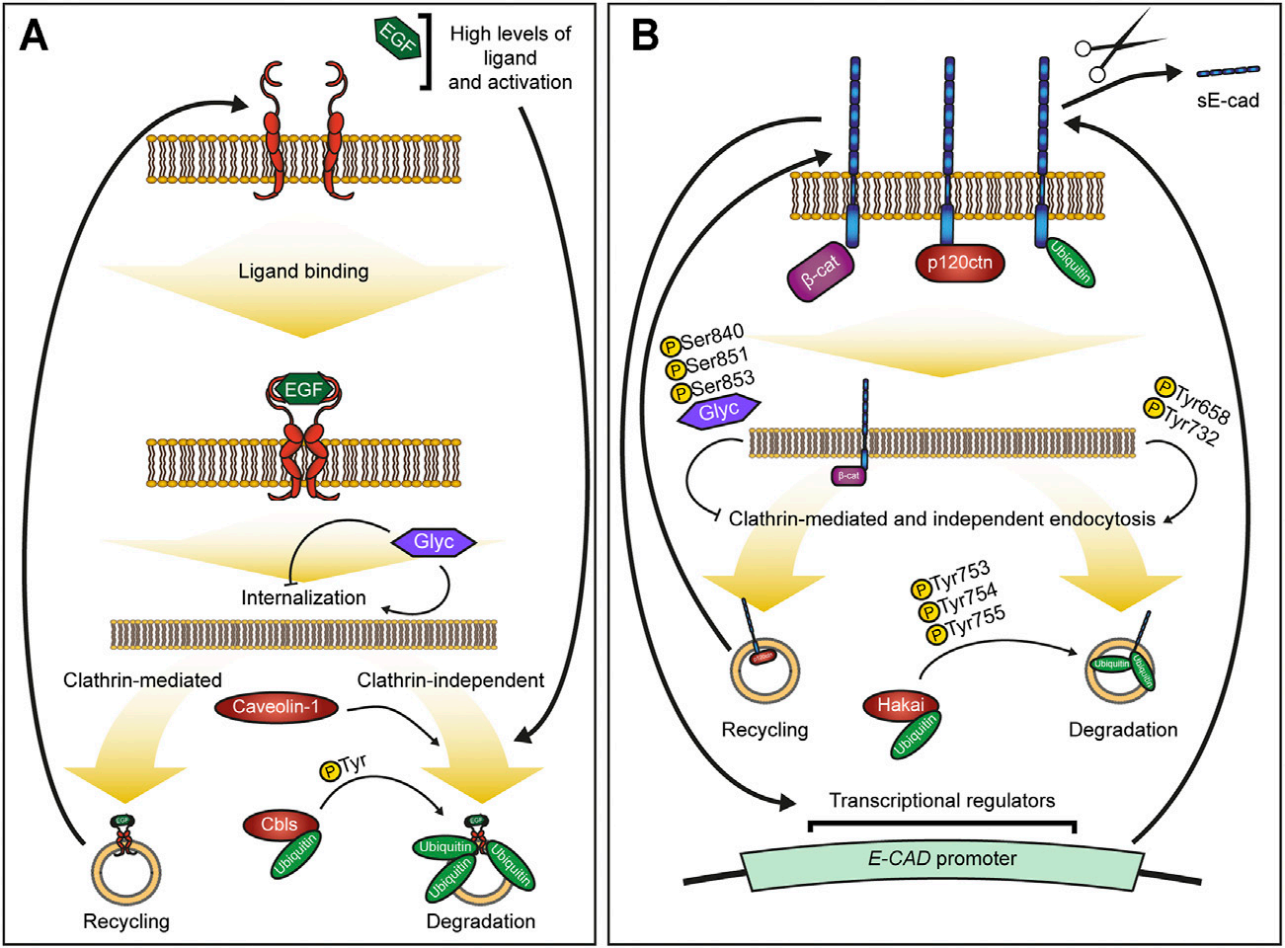
Overview of mechanisms controlling EGFR and E-cadherin endocytosis. **(A,B)** – Summary of EGFR **(A)** and E-cadherin **(B)** regulation. Ligand-induced activation and dimerization of EGFR **(A)** trigger endocytosis of the receptor. The clathrin-mediated endocytosis is followed by recycling of the receptor and comprises most of the endocytic events, whereas clathrin-independent endocytosis, including caveolae, leads to EGFR degradation and is promoted upon a certain threshold of EGFR activation. Ubiquitination by Cbls ubiquitin ligases serves as a key cue for EGFR degradation and is modulated by EGFR phosphorylation. Levels of E-cadherin at the plasma membrane **(B)** are regulated by endocytosis, which is modulated by E-cadherin interactions with its binding partners. β-catenin (β-cat) helps retain E-cadherin at the membrane, whereas p120-catenin (p120ctn) prevents E-cadherin endocytosis for degradation but promotes its recycling. Both clathrin-mediated and independent pathways can be followed by either E-cadherin recycling or degradation, but the latter depends on E-cadherin ubiquitination by the ubiquitin ligase Hakai and potentially others. E-cadherin membrane presentation also regulates own gene (*E-CAD*) expression. Dynamics of both E-cadherin and EGFR is also regulated by glycosylation of their extracellular domains.

EGFR activity is highly dynamic with endocytosis playing a key role in controlling and fine-tuning EGFR signalling (Figure 1A). As it is reviewed elsewhere (see for example (Barbieri et al., 2016; Caldieri et al., 2018; Sigismund et al., 2018)), we will only briefly introduce the main aspects relevant to this minireview. EGFR can be endocytosed through both clathrin-mediated (CME) and independent (CIE) pathways, and the pathway choice is linked with the critical decision for EGFR: its recycling or degradation (Barbieri et al., 2016). The majority of activated EGFR appears to be internalized via CME, which is followed by recycling thus prolonging the signalling (Huang et al., 2004; Sigismund et al., 2008; Rappoport and Simon, 2009; Chi et al., 2011). However, several CIE pathways also contribute to EGFR internalization, including caveolae – smooth vesicles formed by cholesterol- and sphingolipids-rich lipid rafts (Galbiati et al., 2001). This route was found to internalize EGFR at high ligand concentrations in HeLa but not HEp2 cells (Sigismund et al., 2005; Kazazic et al., 2006). Concurrently, lipid rafts and caveolae may prevent EGFR clustering and ligand-independent EGFR activation, which is observed upon cholesterol sequestration and caveolae inhibition with filipin III (Schnitzer et al., 1994; Lambert et al., 2006). Overall, CIE pathways appear to be activated at the high receptor or ligand concentrations and are followed by degradation (Sigismund et al., 2018, 2008). Such response makes physiological sense, promoting EGFR degradation as a countermeasure against hyperactivation (Barbieri et al., 2016). Therefore, it is not surprising that defects in EGFR degradation are seen, for example, in cholangiocarcinoma RBE and breast cancer cells (Gui et al., 2012; Pareja et al., 2012).

Intracellular trafficking of EGFR and its downstream targets are modulated by posttranslational modifications (Figure 1A). Ligand-activated EGFR undergoes dimerization and transautophosphorylation at several residues in the regulatory C-tail, as well as phosphorylation by kinases that act downstream (Miloso et al., 1995; Thelemann et al., 2005; Song et al., 2014; Wee and Wang, 2017). This attunes EGFR interactions, endocytosis and fate but also alters the cellular response to EGFR activation (Tong et al., 2009; Jones and Rappoport, 2014; Wee and Wang, 2017).

A core cue in determining the EGFR fate is ubiquitination, which is mostly placed by Cbl proteins (Levkowitz et al., 1999; Marmor and Yarden, 2004; Thien and Langdon, 2005; Huang et al., 2007). The ubiquitination depends on the present phosphotyrosines, highlighting feedbacks between receptor activation, endocytosis and posttranslational modifications (Sigismund et al., 2013). Deubiquitination of internalized EGFR promotes its recycling, bypassing the degradation pathway (Liu et al., 2013). A threshold EGFR activation is necessary for ubiquitination, switching from CME to CIE and subsequent degradation (Pinilla-Macua et al., 2017).

Finally, the EGFR extracellular domain is rich in sites whose N-glycosylation affects EGFR signalling in multiple ways. Among other roles, N-glycosylation modifies EGFR folding thus regulating its ligand-binding; modulates endocytosis and intracellular trafficking of EGFR thus adjusting protein surface levels and signalling duration; prevents ligand-independent activation; and creates binding sites for extracellular lectins – galectins –, which contribute to the assembly of supramolecular complexes and limit diffusion of receptors in the plasma membrane (recently reviewed in Porębska et al., 2021).

## Overview of E-Cadherin Adhesion Regulation

The membrane levels of E-cadherin determine adhesion strength, but also cell rearrangements and proliferation within the tissue (Chu et al., 2004; Ciesiolka et al., 2004; Mohan et al., 2018), whereas its loss is a hallmark of invasive carcinomas (Birchmeier and Behrens, 1994; Yu et al., 2019). The most characterized route to control E-cadherin surface levels is endocytosis (Figure 1B). Similar to EGFR, E-cadherin can be internalized by both CME and CIE (reviewed in (Nanes and Kowalczyk, 2012), which can be followed by its recycling or degradation (Le et al., 1999; Bulgakova et al., 2013; Cadwell et al., 2016; Brüser and Bogdan, 2017). The fate of internalized E-cadherin is not ultimately linked to the internalization pathway; CME can be followed by either degradation or recycling (Le et al., 1999; Xiao et al., 2003). Instead, the p120-catenin protein, which directly binds the E-cadherin intracellular domain, might be determining the outcome; while p120-catenin binding prevents E-cadherin CME followed by degradation, it also recruits Numb to promote CME followed by recycling (Ishiyama et al., 2010; Sato et al., 2011).

Posttranslational modifications modulate E-cadherin stability, affinity to binding partners and trafficking (Figueiredo et al., 2013; Brüser and Bogdan, 2017). Phosphorylation at Ser840, Ser851 and Ser853 increases E-cadherin affinity to β-catenin and stabilizes adhesion by preventing E-cadherin endocytosis and degradation (Lickert et al., 2000; Jaggi et al., 2005; McEwen et al., 2014). In contrast, phosphorylation of Tyr658 and Tyr732 of VE-cadherin reduces its binding to β-catenin and p120-catenin, negatively affecting its function (Jeanes et al., 2008; Bertocchi et al., 2012; Chen et al., 2016). Phosphorylation of E-cadherin at Tyr753-755 creates a docking site for the E3 ubiquitin ligase Hakai, and possibly others such as March8 (Fujita et al., 2002; Pece and Gutkind, 2002; Kaido et al., 2009; Kim et al., 2014, p. 8). Hakai promotes E-cadherin degradation and competes with p120-catenin for E-cadherin binding (Hartsock and Nelson, 2012). Moreover, Hakai alongside Src also stabilizes δ-catenin, which promotes E-cadherin processing (Palacios et al., 2005; Kim et al., 2012; Shrestha et al., 2017). Various proteinases including matrix metalloproteinase-2 (MMP-2) and matrix metalloproteinase-9 (MMP-9) – whose high levels correlate metastasis and poor prognosis of multiple cancers – can induce proteolytic cleavage of E-cadherin extracellular domain (Roomi et al., 2009; Li et al., 2017). Upon the cleavage, the extracellular proteolytic fragment (soluble E-cadherin, sE-cad) is released into extracellular space, where it has multiple effects including interfering with E-cadherin adhesion, signalling activities and antitumor immune response (Hu et al., 2016). Additionally, glycosylation of the E-cadherin extracellular domain modulates E-cadherin adhesive function and endocytic turnover (Zhao et al., 2008; Advedissian et al., 2017).

Finally, the regulation of E-cadherin transcription involves a complex network of transcriptional repressors, activators, and epigenetic modifiers (Ramirez Moreno et al., 2021). Among others, the closely related transcriptional repressors SLUG and SNAIL (also known as SNAI2 and SNAI1) directly repress E-cadherin transcription by binding conserved E-boxes in its promoter (Batlle et al., 2000; Cano et al., 2000; Bolós et al., 2003). Consistently, changes in the machinery that modulates its expression often lead to loss of E-cadherin in cancers (Bringuier et al., 1999; Bruner and Derksen, 2018; Ramirez Moreno et al., 2021).

## Regulation of E-Cadherin by EGFR Signalling

Changes to EGFR signalling promote epithelial-to-mesenchymal transition (EMT), at least in part by downregulating E-cadherin. EGFR is overexpressed in 70% of malignant ovarian tumours and 85% of salivary adenoid cystic carcinomas, leading to increased mRNA levels of *SLUG* (Bartlett et al., 1996; Cheng et al., 2012, 2013; Wang et al., 2018). In ovarian cancer cells, EGFR activation promotes *SLUG* transcription by inducing the expression of the transcription factor Egr-1, which directly binds to the *SLUG* promoter (Cheng et al., 2013). The relevance of elevated SLUG expression remains controversial: while inhibiting SLUG expression in ovarian SKOV3 and OVACR5 and oviductal OE-E6/E7 cells restored E-cadherin expression and limited cell invasiveness, silencing SLUG did not inhibit EMT in salivary adenoid cystic carcinoma cells (Cheng et al., 2012, 2013; Wang et al., 2018). In ovarian cancer cells SKOV3 and OVCAR3, EGFR activation also increased *SNAIL* mRNA levels, which required EGF-induced H_2_O_2_ production and p38 MAPK activation (Cheng et al., 2010). In contrast, in salivary adenoid cystic carcinoma cells, EGF-induced EGFR activation lincreases levels of SNAIL protein without altering its mRNA levels (Cheng et al., 2012, 2013; Wang et al., 2018). In both cases, however, silencing SNAIL reduced EMT and invasiveness (Cheng et al., 2010; Wang et al., 2018). Curiously, in oviductal epithelial cells, EGFR activation alters neither mRNA nor protein levels of SNAIL (Cheng et al., 2012).

**FIGURE 2:**
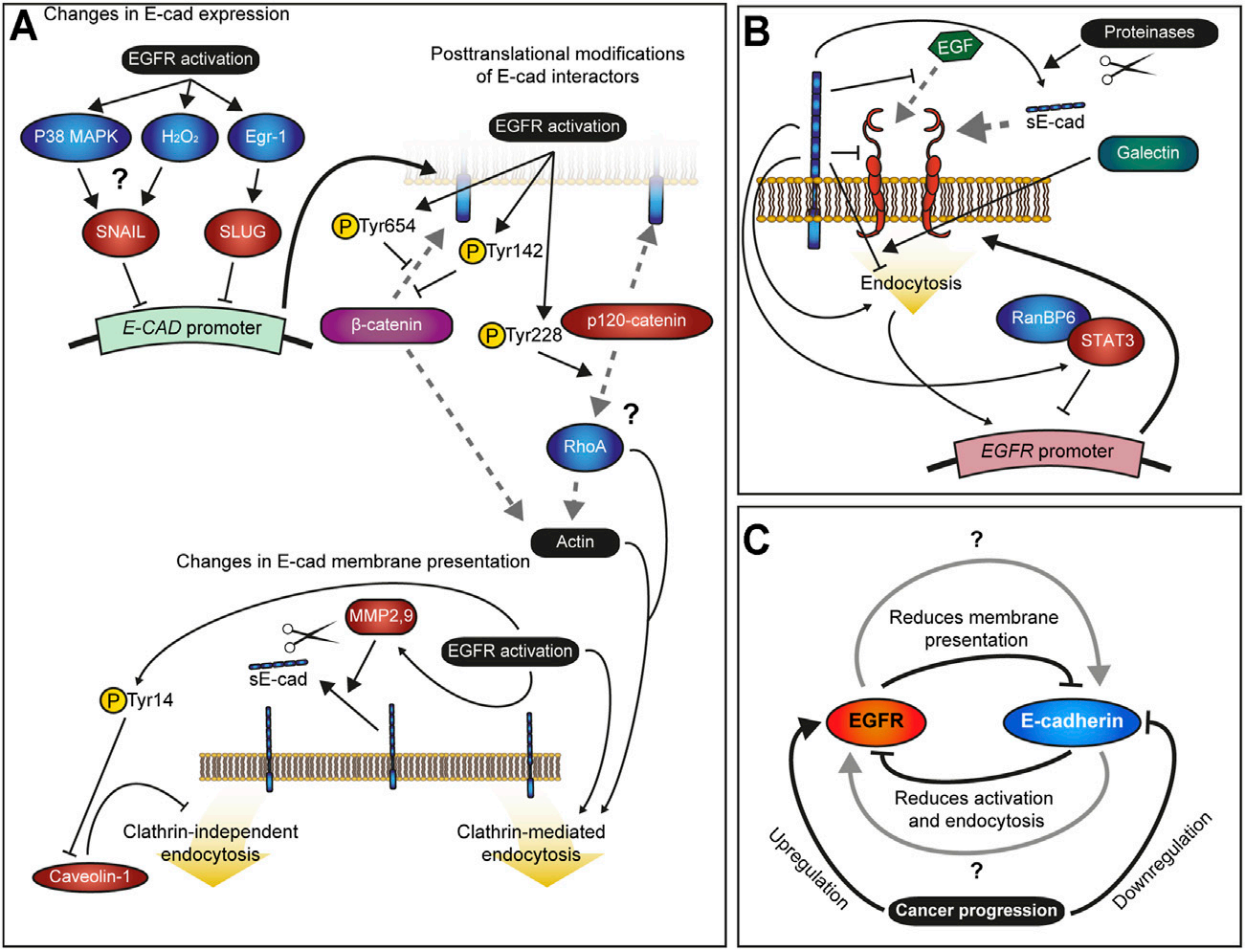
Interactions between EGFR and E-cadherin. **(A)** – Summary of the known effects of EGFR activation on E-cadherin. EGFR signalling downregulates *E-cadherin (E-CAD)* gene expression via the transcriptional repressors SNAIL and SLUG. By promoting phosphorylation, it also destabilizes membrane E-cadherin by reducing its affinity with β-catenin and the subsequent connection to the actin cytoskeleton as well as alters interactions between p120-catenin with RhoA. Additionally, EGFR signalling increases E-cadherin endocytosis by blocking Caveolin-1 activity, a negative regulator of the EGFR pathway itself, and promotes processing of E-cadherin into soluble E-cadherin (sE-cad) through activation of the Matrix Metalloproteinases (MMPs) 2 and 9. Grey dashed lines indicate protein binding. **(B)** – Summary of the known effects of E-cadherin on EGFR. E-cadherin stabilizes EGFR at the membrane, blocks its activation by EGF and reduces its internalization. Through STAT3 and RanBP6, E-cadherin represses *EGFR* gene expression. Additionally, sE-Cad is an agonistic ligand of EGFR, and therefore E-cadherin cleavage, which is promoted by EGFR activity, positively regulates EGFR signalling. **(C)** – Model of the feedback mechanism between EGFR activity and E-cadherin; the negative feedback loop between the two leads to stable EGFR activation and loss of E-cadherin in cancer cells. Gray arrows indicate unknown mechanisms by which both transmembrane proteins coexist and fine-tune each other in normal tissue during development and homeostasis.

EGFR activation also downregulates E-cadherin through several posttranscriptional mechanisms (Figure 2A). Active EGFR induces phosphorylation of both β- and p120-catenin (Hoschuetzky et al., 1994; Hazan and Norton, 1998; Mariner et al., 2004). Phosphorylation of β-catenin at Tyr654 and Tyr142 reduces its affinity for E-cadherin and α-catenin binding, respectively (Roura et al., 1999; Piedra et al., 2003), which could be responsible for the dissociation of E-cadherin from actin cytoskeleton following EGF treatment in breast cancer cells MDA-MB-468 (Hazan and Norton, 1998). EGFR-mediated weakening of association between E-cadherin and actin cytoskeleton may contribute to normal development by enabling cell rearrangements through remodelling of cell contacts, but also promote EMT in malignancy through fragmentation of adherens junctions and cortical actin bundle (Zhitnyak et al., 2020; Fu et al., 2021).

Similarly, EGFR promotes tyrosine phosphorylation of p120-catenin at Tyr228 (Mariner et al., 2004), although the exact intermediate of this phosphorylation is unclear (Alemà and Salvatore, 2007). This residue is present in both common isoforms of p120catenin – mesenchymal isoform 1 and epithelial isoform 3 (Reynolds and Roczniak-Ferguson, 2004). While Tyr228 phosphorylation does not affect p120-catenin binding to E-cadherin and its endocytosis directly, it increases p120-catenin affinity for RhoA binding (Mariner et al., 2004; Castaño et al., 2007; Kourtidis et al., 2013). The effect of this phosphorylation appears to be context-dependent. In E-cadherin-deficient breast cancer cells MDA-MB-231, the binding of p120-catenin N-terminus inhibits RhoA activity (Yanagisawa et al., 2008). Conversely, deletion of the p120-catenin N-terminus inhibits EGF-induced motility, whereas ectopic expression of full-length p120-catenin promotes cell motility in keratinocytes through activation of RhoA and cytoskeletal rearrangements (Cozzolino et al., 2003). This discrepancy in effects of p120-catenin N-terminus on RhoA activity and cell behaviour is consistent with the differences in levels and roles of Tyr228 phosphorylation of p120-catenin in cancer cells. In colon adenocarcinoma cells, phosphorylation of Tyr228 correlates with better prognosis and inhibits cell invasion (Ding et al., 2019), whereas in the breast cancer cells MDA-MB-231 it is essential for the invasiveness-promoting activity of p120-catenin isoform 1 (Yanagisawa et al., 2008; Kourtidis et al., 2015). In either case, the changes in RhoA activity are likely to alter (promote or inhibit) E-cadherin endocytosis depending on the context (Lee and Harris, 2013; Kourtidis et al., 2015; Greig and Bulgakova, 2020).

EGFR activation promotes E-cadherin endocytosis through various routes. In MCF-7 cells, stimulation with EGF promotes either macropinocytosis of E-cadherin or its endocytosis mediated by the small GTPase Arf6, which is likely to be clathrin-dependent (Bryant et al., 2007; Kon et al., 2008). Here, the internalization route might depend on the levels of E-cadherin expression. EGFR activation by EGF also leads to E-cadherin internalization in A431 epidermoid carcinoma cells and A549 lung cancer cells via caveolae (Lu et al., 2003). EGFR activation leads to caveolin-1 phosphorylation at Tyr14 and its fast redistribution from the plasma membrane (Pol et al., 2000; Orlichenko et al., 2006). Caveolin-1 negatively regulates the caveolae-mediated endocytosis due to its ability to stabilize caveolae association with the plasma membrane (Le et al., 2002; Simón et al., 2020). Consistently, disruption of caveolae using filipin III blocks E-cadherin endocytosis following EGFR activation (Lu et al., 2003). In addition, chronic EGFR activity inhibits mRNA expression of *caveolin-1* (Lu et al., 2003), which is likely to ensure sustained caveolae endocytosis of surface E-cadherin. Curiously, knockdown on caveolin-1 is sufficient to downregulate E-cadherin but also leads to SNAIL overexpression (Lu et al., 2003). While EGF-activated EGFR is not internalized by caveolae (Kazazic et al., 2006), disruption of lipid rafts leads to ligand-independent EGFR activation (Lambert et al., 2006). Therefore, we speculate that the changes in gene expression of SNAIL and E-cadherin following caveolin-1 knockdown might be due to an indirect effect of ligand-independent EGFR activation.

Finally, EGFR activation may further inhibit E-cadherin-mediated adhesion through proteolytic cleavage of E-cadherin. The secretion of matrix metalloproteinase-2 (MMP-2) is enhanced by EGF supplementation in salivary gland pleomorphic adenoma cells (Navarini et al., 2017), whereas in some ovarian cancer cell lines (OVEA6 and OVCA 429 but not DOV13 and OVCA 432) EGFR activation increases the expression of matrix metalloproteinase-9 (MMP-9) (Ellerbroek et al., 1998). Conversely, EGF produced by lymphoma cells inhibits MMP-9 expression in neighbouring stromal cells through induction of Egr-1 expression (Bouchard et al., 2010). Such context-dependency indicates that these effects might be indirect and rely on additional factors present in each case.

To summarize, at least five molecular routes links EGFR activity and E-cadherin (Figure 2A). Altogether, this ensures robust inhibition of E-cadherin-mediated adhesion, promoting EMT and cell migration in cancer.

## Regulation of EGFR Signalling by E-Cadherin

The extracellular domain of E-cadherin directly binds EGFR in both mammalian and fly cells (Dumstrei et al., 2002; Qian et al., 2004). This binding promotes EGFR localization at the sites of E-cadherin-mediated adhesion, but also interferes with EGF binding to EGFR and reduces the mobility of EGFR in the plasma membrane (Figure 2B) (Qian et al., 2004; Rübsam et al., 2017). Consequently, the loss of E-cadherin leads to increased ligand binding to EGFR, but at the same time promotes EGFR mobility which may stimulate EGFR dimerization and further boost its activation (Bremm et al., 2008). As the result, the loss of E-cadherin often observed in cancer cells leads to activation of EGFR signalling, thus, promoting cancer cell dissemination (Takahashi and Suzuki, 1996; Bae et al., 2013). Conversely, in some contexts, E-cadherin may have an opposite effect as the induction of E-cadherin adhesion assembly in HaCat keratinocyte cells and MCF-10A mammary epithelial cells leads to EGF-independent EGFR activation and requires the extracellular domain of E-cadherin (Pece and Gutkind, 2000; Fedor-Chaiken et al., 2003). Besides EGF, EGFR can be activated by other ligands (Harris et al., 2003; Singh et al., 2016), including sE-cad (Brouxhon et al., 2014; Hu et al., 2016). Moreover, in MCF7 and MDA-MB-231 breast cancer cell lines sE-cad shows a stronger effect than EGF, and acts additively with it (Brouxhon et al., 2014).

One of the possible, though unexplored, explanations for the observed opposite effects of E-cadherin on EGFR, is its potential effect on EGFR endocytosis (Figure 2B). As described above, endocytosis of EGFR is a powerful mechanism of tuning its activity. Indeed, increased activation of EGFR in cells expressing the E-cadherin mutant, which lacks the exon 8 in its extracellular domain (corresponding to the E-cadherin ectodomain 2) but still binds EGFR, is accompanied by the decreased internalization of EGFR from the plasma membrane upon EGF stimulation (Bremm et al., 2008), indicating that the ectodomain 2 promotes EGFR endocytosis. In contrast, the ectodomain 3 is connected to EGFR by galectin-7, which negatively regulates EGFR endocytosis (Proux-Gillardeaux et al., 2021). Thus, E-cadherin extracellular domain may promote or inhibit EGFR endocytosis depending on the context.

In addition to regulation of EGFR activity at the cell surface, E-cadherin downregulation leads to EGFR upregulation on mRNA level in cells from squamous cell carcinoma of the head and neck (Wang et al., 2011). This upregulation might be an indirect effect of positive feedback whereby EGFR activation at the plasma membrane results in increased expression of the *EGFR* gene (Clark et al., 1985; Oldrini et al., 2017). This feedback was suggested to act to restore levels of EGFR following its activation, internalization and consequent degradation, therefore ensuring the robustness of EGFR signalling (Oldrini et al., 2017). The feedback from EGFR to its own gene expression involves the signal transducer and activator of transcription 3 (STAT3) protein. STAT3 binds *EGFR* promoter and inhibits its transcription in RanBP6-dependent manner in HEK-293T human kidney cells. When demand arises this inhibition can be lifted, for example when additional production of EGFR is required following its ligand-induced degradation (Oldrini et al., 2017). At the same time, inhibition of STAT3 phosphorylation is sufficient to increase levels of *EGFR* mRNA (Oldrini et al., 2017), whereas E-cadherin promotes STAT3 activation in mouse embryonic stem cells (del Valle et al., 2013), suggesting that it can contribute to this feedback.

Therefore, E-cadherin in most cases inhibits EGFR through a combination of modulating its behaviour at the cell surface and promoting transcriptional silencing (Figure 2B). However, E-cadherin acts in more than one way and in some contexts, may activate EGFR instead.

## Conclusion

Both EGFR and E-cadherin are vital for normal development, highly dynamic and often dysregulated in cancer cells. In the latter, there is feedback between the two proteins; EGFR downregulates E-cadherin through multiple mechanisms and vice versa (Figures 2A,B). Such feedback should lead to fast amplification of adhesion loss and EGFR activation, promoting invasiveness and proliferation of a tumour (Figure 2C). However, if the interaction between the two proteins were limited to this feedback, it would be impossible for simultaneous E-cadherin-mediated adhesion and EGFR signalling in a cell. Meanwhile, multiple examples of such cells exist. Human skin stem cells require EGFR activity for proliferation and express E-cadherin, even if at lower levels than other keratinocytes (Molès and Watt, 1997; Brechbuhl et al., 2014). Similarly, during *Drosophila* wing development EGFR activity is required for specification of veins and leads to a basal shift in E-cadherin localization without adhesion loss (O’Keefe et al., 2007). We speculate that expression levels and endocytic trafficking of both proteins play an important role in their effects on each other, as well as the mechanical environment of the cells. Thus, upon mechanical stress, EGFR promotes E-cadherin-mediated cell stiffening through activation of the Abl kinase, leading to the recruitment of vinculin to the adhesion sites (Sehgal et al., 2018). Discovering molecular mechanisms of how EGFR activity and E-cadherin-mediated adhesion co-exist in normal tissues is essential for understanding the causes of the amplifying feedback between them in cancer cells and developing approaches to break this feedback.
